# Gallocatechin Gallate Inhibits the Replication of Pseudorabies Virus via Suppressing the Entry and Release Stages in Its Replication Cycle

**DOI:** 10.3390/vetsci10030189

**Published:** 2023-03-01

**Authors:** Zongyi Bo, Jinjin Zhu, Mengjiao Guo, Chengcheng Zhang, Yongzhong Cao, Xiaorong Zhang, Yantao Wu

**Affiliations:** 1 Joint International Research Laboratory of Agriculture and Agri-Product Safety, The Ministry of Education of China, Yangzhou University, Yangzhou 225009, China; 2 Jiangsu Co-Innovation Center for the Prevention of Animal Infectious Disease and Zoonoses, College of Veterinary Medicine, Yangzhou University, Yangzhou 225009, China

**Keywords:** pseudorabies virus, herpesvirus, anti-PRV compound, gallocatechin gallate, replication cycle, entry stage, release stage

## Abstract

**Simple Summary:**

The development of antiviral compounds is an important method for preventing virus infection. The pseudorabies virus (PRV), one kind of alpha-herpesvirus, which can induce lytic and latent infection in pigs, causes huge economic losses to the Asian swine industry. Not only pigs but also multiple species of infection hosts of PRV have been reported, such as horses, bats, wolves, mice, cattle, etc. More crucially, it has been shown that the pseudorabies virus might be transmitted to human beings from infected animals. However, anti-PRV drugs have been rarely studied. In this study, a library of natural compounds was screened using an EGFP-labeled PRV, and the results showed that gallocatechin gallate, which is an extract from green tea, could significantly inhibit the replication of PRV in a dose-dependent manner. Finally, it was demonstrated that the entry and release stages of the PRV replication cycle were significantly suppressed by gallocatechin gallate. This study offers a new venture for the development of treatments against pseudorabies disease.

**Abstract:**

The pseudorabies virus is a widespread swine pathogen that has caused significant economic losses to the global pig industry. Due to the emergence of PRV variant strains in recent years, vaccines cannot provide complete protection against the infection of PRV. Therefore, the research on antiviral compounds is of great importance for PRV treatment. In this study, an EGFP-labeled PRV was used to screen anti-PRV compounds from 86 natural product extracts. Gallocatechin gallate was found to efficiently inhibit the replication of PRV with a half-maximal inhibitory concentration (IC_50_) of 0.41 μM. In addition, it was found that gallocatechin gallate was unable to directly inactivate PRV and had no effect on the attachment stage of PRV. However, it was found that gallocatechin gallate significantly suppressed the viral entry stage. Furthermore, it was found that the release stage of PRV was also significantly suppressed by gallocatechin gallate. Together, this study found that gallocatechin gallate could efficiently inhibit the replication of PRV by suppressing the entry and release stages of PRV, which will contribute to the development of a new therapeutic strategy against PRV infection.

## 1. Introduction

Pseudorabies disease, or Aujeszky’s disease, was first described by Aujeszky in Hungary [1,2]. Pseudorabies virus (PRV), the causative agent of pseudorabies disease, belongs to the family Herpesviridae, subfamily alpha-herpesvirus, and it is a large double-stranded DNA with a genome of up to 150 kb [3]. PRV causes abortions and stillbirths or mummified fetuses in sows, central nervous system disease in young piglets, and respiratory disease in older pigs [4]. PRV has been eliminated from pigs in many countries [5,6,7]. However, pseudorabies disease is still an important swine disease that causes huge economic losses to the swine industry in Asian areas, especially in China [8,9].

One of the key strategies for the prevention and eradication of pseudorabies disease is vaccination. However, since 2011, there has been a large number of outbreaks of pseudorabies in Bartha-K61 vaccine-immunized swine farms caused by a variant strain in China [10,11,12]. Several studies have demonstrated that the classical Bartha-K61 vaccine could not provide full protection for pigs from infection by PRV variant strains [13,14]. More significantly, it has been a long history of PRV case reporting in humans as early as 1914, which reflects that PRV might be a zoonotic virus [15]. To date, an increasing number of PRV cases in humans have been reported, especially since the outbreak of PRV variant strains in China [9]. The main clinical signs in humans caused by PRV include fever, encephalitis, endophthalmitis, and headaches. So far, no human beings have died from PRV [16]. Therefore, research on effective antiviral drugs is not only important for the prevention and cure of pseudorabies disease in swine but also of great significance to human public health.

Numerous natural compounds have been shown to have antiviral effects in recent years, particularly during the pandemic of COVID-19. To date, there are several anti-PRV compounds have been reported. Resveratrol has a strong inhibitory effect on PRV replication, and the inhibition of virus proliferation by resveratrol is not due to the direct inactivation or inhibition of virus entry into the host cell but rather to the inhibition of virus proliferation in the host cell [17]. Isobavachalcone (IBC) is a naturally occurring chalcone extracted from the tonic, that has a variety of biological functions, including antibacterial, antifungal, and anticancer activities. Recently, IBC was found to have a strong inhibitory effect on PRV in vitro [18]. The anti-PRV effect of meclizine was observed in mouse experiments, and the results showed that meclizine reduced the severity of clinical symptoms and the viral load in tissues after PRV attack and delayed the death of mice [19].

In this study, 86 natural products were screened for anti-PRV compounds using an EGFP-labeled PRV, and the results showed that gallocatechin gallate could efficiently inhibit the replication of PRV, with an IC_50_ of only 0.41 μM. Moreover, it was demonstrated that gallocatechin gallate could significantly inhibit the entry and release stages in PRV replication cycles but had no effect on the attachment stage of PRV. This study found a novel natural product extract, gallocatechin gallate, which can efficiently inhibit PRV replication and provides an alternative choice for the therapy of pseudorabies disease.

## 2. Materials and Methods

### 2.1. Cells and Viruses

PK15 cells were kept in our laboratory and cultured in Dulbecco Modified Eagle Medium (11965092; DMEM, Thermo Fisher Scientific, Waltham, MA, USA) containing 10% fetal bovine serum (S711-001; Lonza, Uruguay) and 1% penicillin/streptomycin (15070063; Thermo Fisher Scientific, Waltham, MA, USA) in a 5% CO_2_ incubator at 37 °C. The PRV JSY13 variant strain (MT157263.1) was kept in our laboratory. 

### 2.2. Antibodies and Reagents

Mouse anti-PRV-UL42 and anti-PRV-UL29 polyclonal antibodies were generated in our laboratory. Rabbit anti-actin (23660-1-AP), HRP-conjugated goat anti-rabbit IgG (SA00001-2), and HRP-conjugated goat anti-mouse IgG (SA00001-1) were purchased from Proteintech (Wuhan, China). Alexa 488-conjugated goat anti-mouse IgG antibody was purchased from Millipore (62197; Billerica, MA, USA). The natural product library containing 86 kinds of natural product extracts (listed in Table S1) was purchased from Selleck (L1400; Houston, TX, USA). Gallocatechin gallate (S9058), one of the compounds in this natural product library, is an extract from green tea, and its degree of purification is 98%. TMB solution was purchased from Solarbio (PR1210; Beijing, China). Enhanced Cell Counting Kit-8 (CCK-8) reagent was purchased from ApexBio (K1018; Houston, TX, USA). 

### 2.3. Anti-PRV-Compound Screening

PK15 cells were preseeded in 96-well plates at 2 × 10^4^ cells/well and incubated at 37 °C overnight. After discarding the culture medium, the cells were pretreated with different compounds at a concentration of 10 μM in DMEM (10% FBS) for 2 h, respectively. The cells were infected with JSY13-EGFP (0.15 MOI), a recombinant of JSY13 with EGFP expression cassette (CMV-EGFP-Poly A) inserted at the place of the TK gene using the homologous recombinant method, and incubated at 37 °C for 18 h. The fluorescence intensity of each well was monitored with a U-HGLGPS microscope (Olympus, Tokyo, Japan).

### 2.4. Cell-Based ELISA

PK15 cells were preseeded in 96-well plates at a density of 2 × 10^4^ cells/well and incubated at 37 °C overnight. The cells were pretreated with different concentrations of gallocatechin gallate and infected with JSY13 at 0.15 MOI for 24 h. Then, the supernatant was discarded, and the cells were gently washed with PBS and fixed in fixation solution (PBS containing 4% paraformaldehyde and 0.1% Triton X-100) at 37 °C for 30 min. The cells were washed three times with glycine-PBS (PBS containing 20 mM glycine) and blocked with PBST containing 3% skimmed milk at 37 °C for 2 h. The cells were incubated with antibodies against PRV-UL42 polyclonal antibody (1:7000) for 2 h at 37 °C. The cells were washed three times with PBST and incubated with HRP-conjugated goat anti-mouse IgG secondary antibody (1: 7000) for 1 h at 37 °C. Both primary and secondary antibodies were diluted with PBS. After washing with PBST 5 times, the cells were incubated with TMB solution for 15 min. Finally, the absorbance value was read at 450 nm.

### 2.5. Western-Blot

PK15 cells were preseeded in 6-well plates at a density of 70% and incubated overnight at 37 °C. The cells were pretreated with different concentrations of gallocatechin gallate and incubated for 2 h. Then, the cells were infected with JSY13 (0.15 MOI) for 24 h. The cells were washed with PBS three times, and cell lysates were collected using 2 × SDS sample buffer (100 mM Tris-Cl (pH 6.8), 20% glycerol, 4% SDS, 0.12% bromophenol blue, and 10% β-Mercaptoethanol). The cell lysates were boiled at 100 °C for 10 min, separated by SDS-PAGE, and transferred onto nitrocellulose (NC) membranes (Pall Corporation, Mexico). The membranes were blocked in 5% skimmed milk in TBST for 2 h at room temperature and incubated with primary antibody overnight at 4 °C. After washing three times with TBST, the membranes were incubated with the respective secondary antibodies for 4 h at 4 °C. The bands were visualized using a high-sensitivity ECL chemiluminescence kit (NCM Biotech, Suzhou, China) via a Tanon 5200 system (Tanon, Shanghai, China).

### 2.6. Indirect Immunofluorescence Assay

Coverslips were placed in 6-well plates and PK15 cells were then seeded on coverslips with 60% density and incubated overnight. The cells were pretreated with different concentrations of gallocatechin gallate for 2 h. Then, the cells were infected with JSY13 (0.15 MOI) for 18 h. Then, the cells were washed with PBS three times and fixed in fixative solution (PBS containing 4% paraformaldehyde and 0.1% Triton X-100) for 30 min at 37 °C. The cells were washed three times with Glycine-PBS (PBS containing 20 mM Glycine) and then blocked with PBST containing 3% bovine serum albumin (BSA) for 30 min at 37 °C. The cells were incubated with anti-PRV UL42 polyclonal antibody (1:500) for 1 h at 37 °C, washed three times with PBS, and then incubated with FITC-conjugated goat anti-mouse IgG secondary antibody (1:500) for 1 h at 37 °C. The cells were washed three times with PBS, and the nuclei were stained with DAPI (Beyotime, Shanghai, China) at room temperature. Finally, the cells were visualized with U-HGLGPS microscopes (Olympus, Tokyo, Japan).

### 2.7. The mRNA Level of PRV Genes and GAPDH Was Measured Using qPCR

Total RNA was extracted from cells using an RNA extraction kit (CwBiotech, Suzhou, China) according to the manufacturer’s instructions, and the RNA concentration was determined. Then, the RNA was reverse transcribed into cDNA using the reverse transcription reagent EasyScript Reverse Transcriptase (TransGen, Beijing, China). Fluorescent qPCR qMix (Vazyme, Nanjing, China) was used according to the manufacturer’s recommendations. Quantitative PCR (qPCR) was performed on a LineGene 9600 Plus Fluorescent Quantitative Polymerase Reaction (PCR) Detection System (Bioer Technology, Hangzhou, China). The thermal profile for qPCR was set as 95 °C for 5 min and then 35 cycles of 95 °C for 15 s, 56 °C for 15 s, and 72 °C for 30 s. The GAPDH gene was amplified with the sense primer, 5′-CCTTCATTGACCTCCACTACA-3′; and the antisense primer, 5′-GATGGCCTTTCCATTGATGAC-3′. The PRV-UL48 gene was amplified with the sense primer, 5′-GTTCATGTCCCTGCGCTAC-3′, and the antisense primer, 5′-CACCTGGTGCGAGAGGTAGC-3′.

### 2.8. Detection of the Effect of Gallocatechin Gallate on the Attachment of PRV

PK15 cells were preseeded in 6-well plates at 7 × 10^5^ cells/well, and 16 h later, the cells were pretreated with gallocatechin gallate (2 μM) in DMEM containing 10% FBS for 2 h. Then, the culture was discarded, and the cells were treated with serum-free DMEM containing 5 MOI JSY13 and 2 μM gallocatechin gallate at 4 °C for 1 h. The cells were then washed three times with cold PBS, 1 mL of TRIzol was added, total RNA was extracted and reverse transcribed into cDNA, and the mRNA levels of GAPDH and PRV-UL48 were determined using qPCR. For the virus titer assay, 1 mL serum-free DMEM was added, and the cells were subjected to three freeze–thaw cycles before the plaque formation assay was performed as described [20].

### 2.9. Detection of the Effect of Gallocatechin Gallate on PRV Entry into Cells

PK15 cells were preseeded in 6-well plates at 7 × 10^5^ cells/well overnight, washed with PBS three times, and infected with JSY13 (5 MOI) in serum-free DMEM at 4 °C for 1 h. The cells were washed three times with PBS, and 1 mL DMEM with 2% FBS containing 2 μM gallocatechin gallate was added to the cells and incubated at 37 °C for 1 h. The cells were washed three times with sodium citrate (pH 3.0) and then washed three times with PBS. Then, 1 mL of TRIzol was added, and total RNA was extracted and reverse transcribed into cDNA, and the changes in GAPDH and PRV-UL48 were measured by qPCR. For the plaque formation assay, 1 mL of serum-free DMEM was added. For the virus titer assay, 1 mL serum-free DMEM was added, and the cells were subjected to three freeze–thaw cycles before a plaque formation assay was performed.

### 2.10. Assaying the Effect of Drugs on PRV Release

PK15 cells were preseeded in 6-well plates at 7 × 10^5^ cells/well overnight. The cells were infected with JSY13 (1 MOI) in serum-free DMEM and incubated at 37 °C for 1 h. Then, the cells were washed with PBS three times, and 1 mL DMEM containing 2% FBS was added and incubated at 37 °C for 2 h. The culture medium was discarded, and the cells were treated with gallocatechin gallate (2 μM) in 1 mL DMEM containing 2% FBS and incubated at 37 °C for 1 h. The supernatant was collected to determine the virus titer using a plaque formation assay, and the virus titer in the cells was also determined.

### 2.11. Assay for Direct Inactivation of PRV by Gallocatechin Gallate

JSY13 virus stock solution (200 μL) was treated with 2 μM gallocatechin gallate at 37 °C for 1 h. A plaque formation assay was used to compare the viral titer of gallocatechin gallate-treated or -untreated viruses. 

### 2.12. Cell Viability Assay

Using the CCK-8 reagent, the viability of PK15 cells after gallocatechin gallate treatment was determined as previously reported [21]. PK15 cells were preseeded in 96-well plates at 2 × 10^4^ cells/well. The cells were then treated with different concentrations of gallocatechin gallate. After 24 h, CCK-8 solution (10 μL) was added to the cells and incubated for 1–4 h. To determine the viability of the cells, the absorbance at 450 nm was measured and the ratio of cells treated with gallocatechin gallate to cells treated with DMSO was computed.

### 2.13. Statistical Analysis

GraphPad Prism 8.0 (GraphPad Software, San Diego, CA, USA) was used for the statistical analyses. All data were collected from three independent experiments; the data were expressed as the mean ± standard deviation and analyzed with Student’s *t*-test among different groups. Significance in all figures is indicated as follows: *, *p* < 0.05; **, *p* < 0.01; ***, *p* < 0.001.

## 3. Results

### 3.1. Fluorescence-Based Screening of the Anti-PRV Compounds 

**Figure 1:**
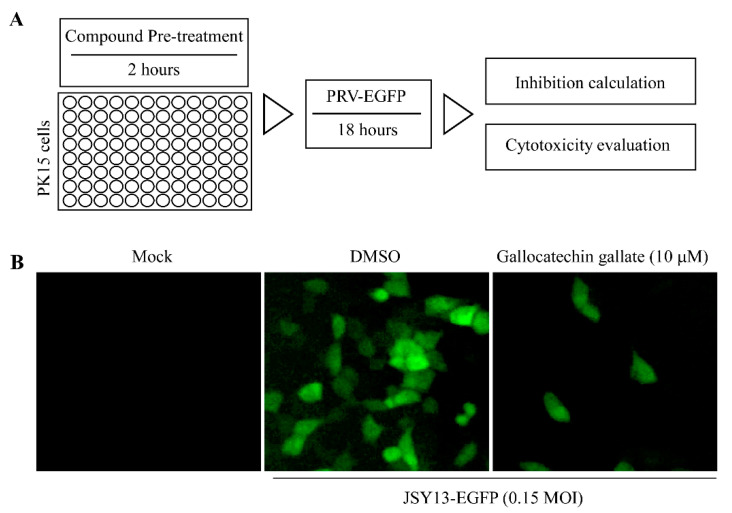
Gallocatechin gallate efficiently inhibited the replication of PRV. (**A**) Schematic of the processes in the screening of anti-PRV compounds. The cells in 96-well plates were pretreated with different kinds of compounds (10 μM) for 2 h. The cells were then infected with JSY13-EGFP (0.15 MOI), and 18 h later, the fluorescence intensity of each well was evaluated. (**B**) Gallocatechin gallate inhibits the replication of PRV. The experiment was performed as introduced in (**A**), and the fluorescence figure in gallocatechin gallate-treated cells was photographed.

To screen the effective anti-PRV compounds, an EGFP-inserted PRV JSY13 strain constructed in our previous study was used as a marker of the PRV replication. In this study, the cells in 96-well plates were pretreated with the compounds at a concentration of 10 μM for 2 h, the cells were infected with JSY13-EGFP (0.15 MOI) for 18 h. Then, the fluorescence intensity of each well was evaluated to measure the inhibitory effect of the different compounds on the replication of PRV. The main procedures were performed as shown in Figure 1A. Meanwhile, compounds that had obvious cell cytotoxicity were excluded. The results showed that gallocatechin gallate could efficiently inhibit the replication of PRV (Figure 1B).

### 3.2. Gallocatechin Gallate Inhibits the Replication of PRV with a Low IC_50_ of 0.41 μM

**Figure 2:**
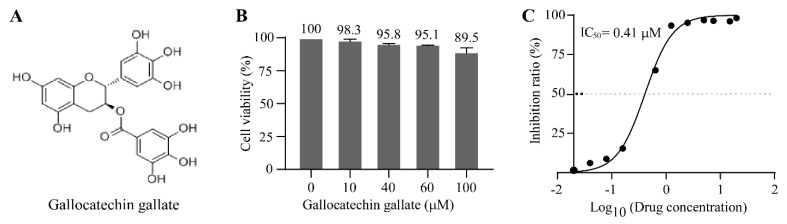
The cell cytotoxicity of gallocatechin gallate and its IC_50_ to the replication of PRV in PK15 cells. (**A**) The chemical formula of gallocatechin gallate. (**B**) PK15 cells in 96-well plates were treated with gallocatechin gallate at concentrations of 10 μM, 40 μM, 60 μM, and 100 μM for 24 h, and then CCK-8 (10 μL) solution was added and incubated for 3 h. The absorbance value at 450 nm was measured, and the results were calculated as the percentage of the OD_450_ of gallocatechin gallate-treated group to the DMSO-treated group. (**C**) The IC_50_ of gallocatechin gallate was 0.41 μM. PK15 cells in 96-well plates were pretreated with gallocatechin gallate at concentrations of 0.02 μM, 0.04 μM, 0.08 μM, 0.16 μM, 0.64 μM, 1.25 μM, 2.5 μM, 5 μM, 7.5 μM, 15 μM, and 20 μM. Two hours later, the cells were infected with JSY13 for 24 h. Then, cell-based ELISA was performed, and the IC_50_ of gallocatechin gallate was determined with GraphPad Prism 8.0 software.

Gallocatechin gallate is a kind of polyphenol (Figure 2A) that is extracted from green tea. To exclude the possibility that the anti-PRV effect was caused by the cytotoxic effect of gallocatechin gallate in PK15 cells, cell viability was measured using CCK-8 reagent. The results showed that the cell viability of the gallocatechin gallate-treated group was 98.3%, 95.8%, 95.1%, and 89.5% at concentrations of 10 μM, 40 μM, 60 μM, and 100 μM, respectively, when they are compared with the DMSO control group (Figure 2B). These results demonstrated that there was no obvious cytotoxicity of gallocatechin gallate, as the concentration of gallocatechin gallate used in our study was no more than 10 μM. To calculate the IC_50_ of gallocatechin gallate, a cell-based ELISA was used in this study. Briefly, PK15 cells were pretreated with different concentrations of gallocatechin gallate, for 2 h, and the cells were infected with JSY13 for 24 h. After virus infection, the cell-based ELISA was performed, and the IC_50_ of gallocatechin gallate was calculated as 0.41 μM (Figure 2C).

### 3.3. Gallocatechin Gallate Inhibits the Replication of PRV in a Dose-Dependent Manner

**Figure 3:**
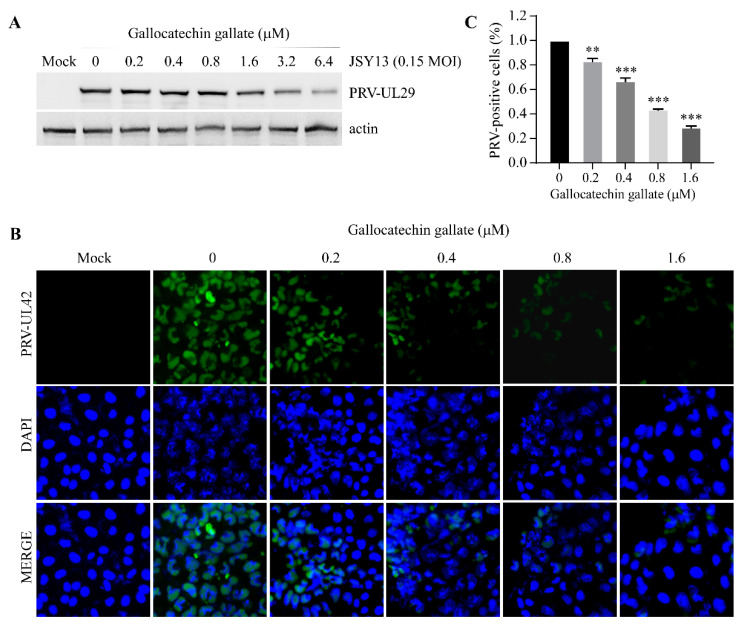
Gallocatechin gallate suppressed the replication of PRV in a dose-dependent manner. (**A**) Gallocatechin gallate could suppress the proliferation of PRV as shown by western-blot assay. PK15 cells were pretreated with 0.2 μM, 0.4 μM, 0.8 μM, 1.6 μM, 3.2 μM, and 6.4 μM gallocatechin gallate for 2 h, and the cells were infected with PRV-JSY13 (0.15 MOI) for 24 h. The cell samples were collected using 2 × SDS buffer and subjected to western blot assay. The expression levels of PRV-UL29 protein were analyzed by immunoblotting. (**B**) Gallocatechin gallate could suppress the proliferation of PRV via IFA assay. PK15 cells were pretreated with 0.2 μM, 0.4 μM, 0.8 μM, and 1.6 μM gallocatechin gallate for 2 h, and then infected with JSY13 (0.15 MOI) for 18 h. After fixation and blocking, the cells were incubated with PRV-UL42 antibody and goat–anti-mouse secondary antibody. Finally, the cells were stained with DAPI and captured with a U-HGLGPS microscope. (**C**) Graph shows the percentage of PRV-positive cells in experiment (**B**) from three independent experiments. (**, *p* < 0.01; ***, *p* < 0.001).

To further confirm the inhibitory effect of gallocatechin gallate on the replication of PRV, PK15 cells were pretreated with gallocatechin gallate at concentrations of 0.2 μM, 0.4 μM, 0.8 μM, 1.6 μM, 3.2 μM, and 6.4 μM. Then, the cells were infected with JSY13 at 0.15 MOI for 24 h. The cell lysates were subjected to immunoblotting experiments, and the results showed that gallocatechin gallate efficiently inhibited the replication of PRV in a dose-dependent manner (Figure 3A). In addition, IFA was performed to evaluate the inhibitory effect of gallocatechin gallate on the replication of PRV. PK15 cells were pretreated with gallocatechin gallate at concentrations of 0.2 μM, 0.4 μM, 0.8 μM, and 1.6 μM. Then, the cells were infected with JSY13 (0.15 MOI), and indirect immunofluorescence assays were performed 18 h post-infection. Similar to the results of the western-blot experiment, the results of the IFA experiment showed that the higher the concentration of gallocatechin gallate was, the more pronounced the inhibitory effect on the replication of PRV (Figure 3B). These results suggested that gallocatechin gallate could effectively inhibit the replication of PRV infection in a dose-dependent manner.

### 3.4. Gallocatechin Gallate Suppressed the Entry Stage of PRV

**Figure 4:**
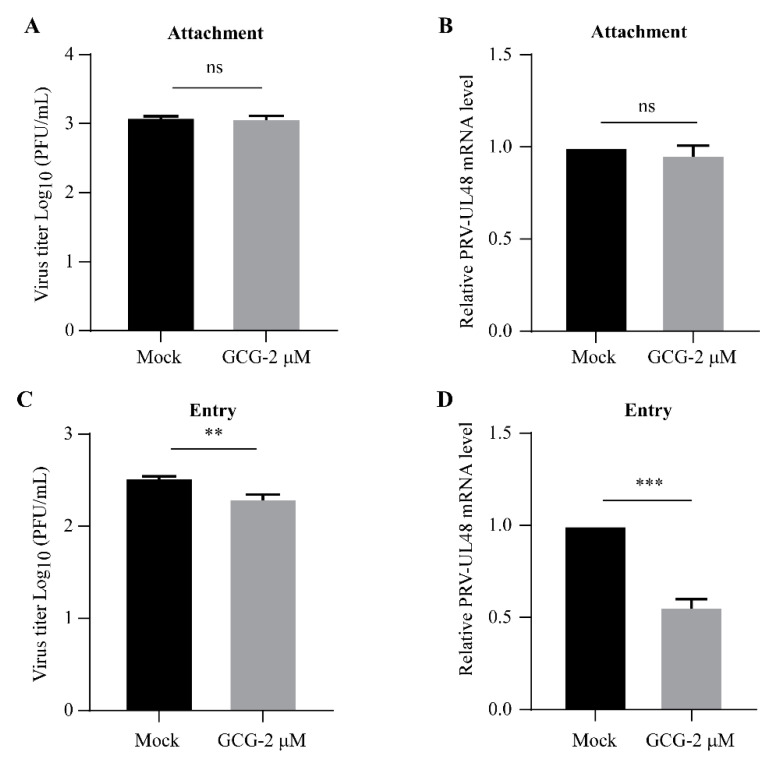
The entry stage in the PRV replication cycle was suppressed by gallocatechin gallate. (**A**,**B**) Gallocatechin gallate had no effect on the attachment stage in the PRV replication cycle. PK15 cells were pretreated with 2 μM gallocatechin gallate for 2 h and then infected with JSY13 (5 MOI) for 1 h at 4 °C. The cell samples were washed with PBS three times, and the cells were collected using 1 mL DMEM and subjected to a plaque formation assay to measure the amount of PRV (**A**). Alternatively, the cells were also collected using TRIzol reagent, and the mRNA level of the PRV-UL48 gene was measured using a qPCR assay (**B**). (**C**,**D**) Gallocatechin gallate suppressed the entry stage in the PRV replication cycle. PK15 cells were infected with PRV (5 MOI) and incubated at 4 °C for 1 h, then the cells were treated with gallocatechin gallate (2 μM) at 37 °C for 1 h. The cells were washed with cold sodium citrate (pH 3.0) three times and then washed with PBS three times. The cells were collected with 1 mL DMEM, and the total amount of PRV was measured using a plaque formation assay (**C**). Alternatively, the cells were also collected using TRIzol reagent, and the mRNA level of the PRV-UL48 gene was measured using a qPCR (**D**). (ns, not significant; **, *p* < 0.01; ***, *p* < 0.001).

The life cycle of PRV mainly consists of four phases: attachment, entry, replication, and release [22,23]. To explore in which stage gallocatechin gallate works, the replication processes were investigated one by one. First, in the attachment assay, PK15 cells were pretreated with gallocatechin gallate for 2 h at 37 °C. Then, the cells were infected with JSY13 (5 MOI) for 1 h at 4 °C, which slowed the entry process and allowed the viruses to stay at the attachment stage. The total amount of the viruses attached to the cells was measured using a plaque formation assay, and the results showed that there was no significant difference between the gallocatechin gallate-treated group and the DMSO-treated group (Figure 4A). Meanwhile, the qPCR assay was used to measure the copy numbers of the viral DNA. The results showed that the mRNA level of PRV-UL48 was not significantly different between the gallocatechin gallate-treated group and the control group (Figure 4B).

In the viral entry analysis, PK15 cells were first infected with JSY13 and incubated at 4 °C for 1 h, and then the cells were treated with gallocatechin gallate at 37 °C for 1 h. The viruses still attached at the cell surface were washed with cold sodium citrate (pH 3.0). The PRV inside the cells was collected and measured using a plaque formation assay. The results showed that the number of viruses in the gallocatechin gallate-treated group was significantly decreased compared to that in the DMSO-treated group (Figure 4C). Meanwhile, the copy number of viral DNA inside the cells was measured using qPCR, and the results showed that the addition of gallocatechin gallate resulted in a significant reduction in the copy numbers of the PRV-UL48 gene (Figure 4D). 

### 3.5. Gallocatechin Gallate Inhibits the Release Stage in the PRV Replication Cycle

**Figure 5:**
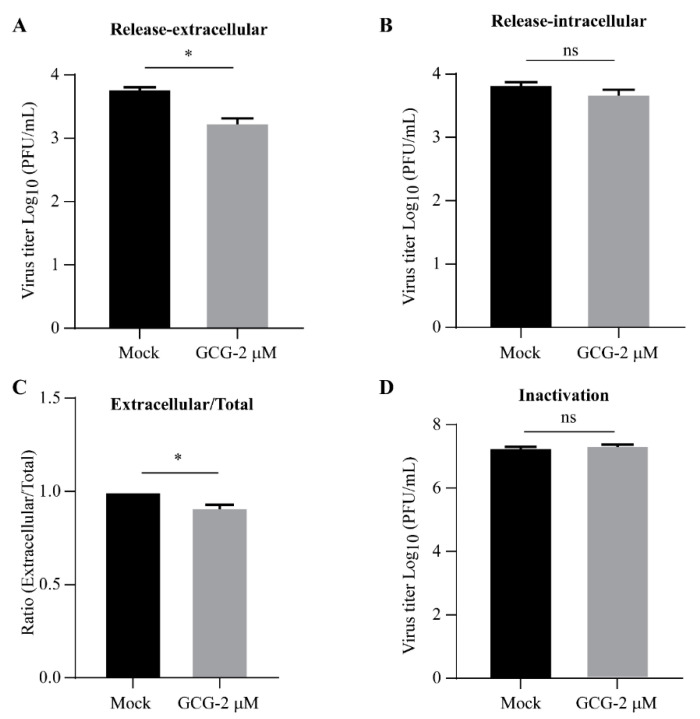
Gallocatechin gallate could suppress the release stage of PRV and was unable to inactivate PRV directly. (**A**–**C**) Gallocatechin gallate inhibited the release of PRV. PK15 cells were infected with JSY13 (1 MOI) in serum-free DMEM and incubated at 37 °C for 1 h. Then, the medium was changed into DMEM containing 2% FBS and incubated at 37 °C for 2 h. The cells were treated with gallocatechin gallate (2 μM) and incubated at 37 °C for 1 h. The viruses in the supernatant (**A**) and cells (**B**) were titered using a plaque formation assay, respectively. (**C**) The ratio of the extracellular PRV to the total amount was calculated to measure the release percentage of PRV. (**D**) JSY13 (200 μL) was mixed with 2 μM gallocatechin gallate and incubated at 37 °C for 1 h, and the virus titer was measured using a plaque formation assay. (ns, not significant; *, *p* < 0.05).

To check whether gallocatechin gallate could affect the release stage of PRV, PK15 cells were infected with JSY13 at 37 °C for 3 h to let the virus finish the attachment and entry stages. After PRV entered the release stage, the cells were treated with gallocatechin gallate for 1 h, and the released viruses in the culture medium and the unreleased viruses in cells were collected and titered separately. The results showed that the released extracellular PRV was significantly decreased (Figure 5A) and the intracellular PRV had no significant difference compared with the control group (Figure 5B). The release ratio was calculated as the extracellular PRV divided by the total amount of extracellular and intracellular PRV, and the results showed that the release ratio was significantly decreased compared with that of the control group (Figure 5C). Taken together, these data demonstrated that gallocatechin gallate could significantly suppress the release stage of PRV.

Finally, to check whether gallocatechin gallate could directly inactivate PRV, JSY13 virus stock solution was added with gallocatechin gallate (2 μM) at 37 °C for 1 h. Then, the virus titer was determined using a plaque formation assay, and the results showed that there was no significant difference in the viral titer after treatment with gallocatechin gallate (Figure 5D).

## 4. Discussion

Since 2011, PRV variant strains have been isolated in China, and traditional PRV vaccines are unable to completely prevent PRV infection in pigs, which brings a great challenge to the prevention and eradication of pseudorabies disease [12,24]. Moreover, similar to other herpesviruses, PRV also has a range wide of infection species, including sheep, cats, coyotes, foxes, rats, deer, bears, rabbits, dogs, horses, bats, wolves, mice, cattle, etc. [25]. In contrast to pigs, other animals infected with PRV die several days after infection [23,26]. Therefore, it is of great importance to explore anti-PRV drugs.

In this study, an EGFP gene-inserted PRV variant JSY13 strain was selected as the target strain, and 86 natural product compounds were selected for anti-PRV compound screening. The replication of PRV will be visible directly using EGFP-labeled PRV, which will contribute to the accuracy of the compound screening. Meanwhile, as some compounds might have cell cytotoxic effects, it is necessary to observe the morphology of the cells. Briefly, the PK15 cells in 96-well plates were pretreated with the compound at the same concentration (10 μM) for 2 h, and then the cells were infected with PRV-EGFP for 18 h to measure the density of fluorescence. After the screening, we found that gallocatechin gallate, an extract from green tea, could efficiently inhibit the replication of PRV in PK15 cells (Figure 1B). Meanwhile, the CC_50_ and IC_50_ were measured using a cell-based ELISA method. The results showed that the cytotoxicity of gallocatechin gallate in PK15 cells was very low, as when the concentration of gallocatechin gallate was 100 μM, the cell viability was still at 89.5% (Figure 2B). Therefore, the CC_50_ of gallocatechin gallate in PK15 cells was hard to measure. In contrast, the IC_50_ assay showed that the IC_50_ value of gallocatechin gallate was as low as 0.41 μM, which demonstrated that gallocatechin gallate was a very effective compound in inhibiting the replication of PRV (Figure 2C). Meanwhile, the inhibitory effect of gallocatechin gallate on the replication of PRV was also confirmed by western blot experiments (Figure 3A) and IFA experiments (Figure 3B), which also demonstrated that gallocatechin gallate could efficiently inhibit the replication of PRV in a dose-dependent manner.

The replication cycle of PRV consists of four main stages: attachment, cell entry, replication, and release [27]. To explore the stage in the PRV replication cycle at which gallocatechin gallate works, the effects of gallocatechin gallate on the attachment, entry, and release stages of PRV were further investigated in this study. Similar to other herpesviruses, PRV infection is initiated by the attachment of virions with heparan sulfate proteoglycans via PRV gC protein [28,29]. First, a plaque formation assay and the qPCR method were used to investigate the effect of gallocatechin gallate on the attachment of PRV by incubating the virus for 1 h at 4 °C, which could slow the entry process of PRV. It was demonstrated that gallocatechin gallate had no effect on the attachment stage (Figure 4A,B). Then, PRV gB, gH, and gL mediate the fusion of the viral envelope with the cytoplasmic membrane, allowing the penetration of the viral capsid into the cytoplasm [30]. It found that gallocatechin gallate could significantly inhibit the entry stage of PRV (Figure 4C,D). The egress of PRV mainly consists of two membrane transport events, the egress of full capsids from the nucleus combined with the mature virion from the cytoplasmic membrane [31,32,33]. In our study, it was demonstrated that gallocatechin gallate could significantly inhibit the release stage of PRV (Figure 5A–C). Finally, to investigate whether gallocatechin gallate directly inactivates PRV, the viruses were incubated with gallocatechin gallate for 1 h, and a plaque formation assay was performed. The results showed that gallocatechin gallate did not directly affect the infectiousness of the PRV virion (Figure 5D). Taken together, we found that gallocatechin gallate could significantly suppress the entry and release stages of PRV, and it was speculated that gallocatechin gallate might affect the membrane fusion between the cells and PRV.

Gallocatechin gallate is a kind of polyphenol derived from green tea (Camellia sinensis), a plant that is widely grown and consumed as a beverage in many countries. It has been reported that gallocatechin gallate has a variety of biological activities, such as antitumor, apoptosis induction, cell cycle arrest, cell growth inhibition, and the production inhibition of α-glucosidase and DPPH [34,35]. In addition, it has been demonstrated that gallocatechin gallate can inhibit the replication of multiple kinds of viruses. For example, it was found that gallocatechin gallate could inhibit the replication of SARS-CoV-2 by disrupting the liquid phase condensation of its nucleocapsid protein [36] and disrupting the enzyme activity of M^pro^ [37]. Another study showed that gallocatechin gallate could inhibit the replication of enterovirus 71 (EV71) by reducing the generation of reactive oxygen species (ROS) [38]. However, this study has demonstrated that gallocatechin gallate could inhibit the replication of PRV via viral entry and release suppression. Whether the biological functions of gallocatechin gallate, such as intracellular lipid droplet reduction, intracellular ROS inhibition, MAPK pathway attenuation, and apoptosis inhibition [39,40], participate in PRV replication is still worthy of further research. In addition, whether gallocatechin gallate is sufficient to prevent the spread of PRV in vivo or alleviate the clinical symptoms of PRV still needs further exploration.

Taken together, this study found a new natural product extract, gallocatechin gallate, that could efficiently inhibit the replication of PRV. The IC_50_ of gallocatechin gallate in PRV inhibition was as low as 0.41 μM. It was also found that gallocatechin gallate had no effect on the attachment stage in the PRV replication cycle and could not inactivate PRV directly. Importantly, it was found that gallocatechin gallate significantly suppressed the viral entry and release stages in the PRV replication cycle. This finding offers a promising therapeutic method against PRV infection.

## Data Availability

Not applicable.

## Supplementary Materials

The following supporting information can be downloaded at: https://www.mdpi.com/article/10.3390/vetsci10030189/s1, Table S1: The list of screened natural product compounds; Origional figures.
